# Bayesian State-Space Modelling of Conventional Acoustic Tracking Provides Accurate Descriptors of Home Range Behavior in a Small-Bodied Coastal Fish Species

**DOI:** 10.1371/journal.pone.0154089

**Published:** 2016-04-27

**Authors:** Josep Alós, Miquel Palmer, Salvador Balle, Robert Arlinghaus

**Affiliations:** 1 Department of Biology and Ecology of Fishes, Leibniz-Institute of Freshwater Ecology and Inland Fisheries, Müggelseedamm 310, 12587 Berlin, Germany; 2 Instituto Mediterráneo de Estudios Avanzados, IMEDEA (CSIC-UIB), C/ Miquel Marqués 21, 07190, Esporles, Illes Balears, Spain; 3 Division of Integrative Fisheries Management, Faculty of Life Sciences, Humboldt-Universität zu Berlin, Invalidenstrasse 42, 10155 Berlin, Germany; Colorado State University, UNITED STATES

## Abstract

State-space models (SSM) are increasingly applied in studies involving biotelemetry-generated positional data because they are able to estimate movement parameters from positions that are unobserved or have been observed with non-negligible observational error. Popular telemetry systems in marine coastal fish consist of arrays of omnidirectional acoustic receivers, which generate a multivariate time-series of detection events across the tracking period. Here we report a novel Bayesian fitting of a SSM application that couples mechanistic movement properties within a home range (a specific case of random walk weighted by an Ornstein-Uhlenbeck process) with a model of observational error typical for data obtained from acoustic receiver arrays. We explored the performance and accuracy of the approach through simulation modelling and extensive sensitivity analyses of the effects of various configurations of movement properties and time-steps among positions. Model results show an accurate and unbiased estimation of the movement parameters, and in most cases the simulated movement parameters were properly retrieved. Only in extreme situations (when fast swimming speeds are combined with pooling the number of detections over long time-steps) the model produced some bias that needs to be accounted for in field applications. Our method was subsequently applied to real acoustic tracking data collected from a small marine coastal fish species, the pearly razorfish, *Xyrichtys novacula*. The Bayesian SSM we present here constitutes an alternative for those used to the Bayesian way of reasoning. Our Bayesian SSM can be easily adapted and generalized to any species, thereby allowing studies in freely roaming animals on the ecological and evolutionary consequences of home ranges and territory establishment, both in fishes and in other taxa.

## Introduction

The home range is defined as the area used by an animal during its normal activities [1]. Establishment of spatially confined home ranges, which may also define an actively defended territory, is a widely observed pattern in nature [2]. The exploration, extension and stability of home ranges have fundamental ecological and evolutionary consequences [3], for example, by determining where predator-prey or intra-specific agonistic interactions occur [4–6]. The mechanistic idea behind the home range concept is that an animal moves following random stimuli (i.e., diffusion movement) but with an added tendency to remain around a specific point, which constrains the fraction of the available potentially suitable habitat to one that is actually used [7,8]. Among the different mechanistic movement models that have been proposed for describing home range behavior in animals, biased random walks are probably the most widespread [9,10]. Describing the drift that constrains the animal around the center of the home range by a bivariate Ornstein—Uhlenbeck (OU) process dates back at least to 1997 [11], and this specific implementation has been repeatedly used since then providing mechanistic descriptors of home range behaviour for a range of wild-living animals (e.g., [12–14]).

Understanding the exact mechanisms driving the establishment of home ranges is not only relevant from a fundamental perspective of behavioral ecology, but can also inform the effectiveness of spatial management actions, such as the design of protected areas [15–18]. However, the methods usually used to obtain positional data in aquatic systems via telemetry suffer from substantial observational error [19,20]. Addressing this methodological issue is crucial to generate reliable inferences about the drivers of the home range establishment in nature. Global positioning systems do not work in aquatic environments. Hence, alternative biotelemetry methods have been proposed for positioning fish and other aquatic animals, such as satellite tracking (e.g., [21–23]). However, the positioning error caused by geolocation in satellite-based biotelemetry applications is usually large (up to several km), which reduces the usefulness of this technology for the fine-scale mechanistic studies of home range behavior in coastal or freshwater fishes thriving in smaller lakes or river sections [24–26].

Alternative acoustic telemetry systems have been developed for the study of the behavior of marine coastal fishes [27]. In such applications, an acoustic transmitter is implanted in the fish that emits a periodic series of ultrasonic pulses that are eventually detected by one or more submerged receivers [28–30]. The standard data that one obtains is a time-series of detections (including of course many missing data) from an array of spatially spaced receivers. Generating precise positions is only possible by examination of multivariate time-series resulting from arrays where hydrophones are located reasonably close to each other. Improved acoustic telemetry systems have recently been designed for high resolution fine-scale behavioral studies based on automated time synchronization of acoustic signals received by various hydrophones (e.g., VPS system from Vemco^®^ or the MAP system by Lotek^®^, [19,31,32]). However, the most commonly used system for learning about movement behavior in marine fish currently consists of arrays of fixed automatic omnidirectional receivers without a fine time synchronization [33].

A detection event occurs when a tagged fish with a transmitter is sufficiently close to the receiver. The fish’s position is still frequently interpolated from the position of the receivers that have detected the fish over a predetermined period of time or time-step [34–36]. However, this procedure may result in biased positioning and may induce incorrect conclusions regarding the characterization of the home range behavior [37–39] or, in general, any other characteristics of fish’ movement [40]. Moreover, there is substantial evidence that the probability of detection is not only function of the distance between a fish and a hydrophone but that it can also be highly influenced by several environmental factors that affect sound propagation in water, such as water currents, tidal phase or environmental noise [41]. All these factors—either isolated or combined—may introduce bias when positioning an animal and they can in turn affect the inference of the mechanisms of home range behavior [42–44]. Therefore, it has been recommended to set acoustics transmitters at known positions within the array (which are known as beacon tags or control tags) for calibrating the environmental effects on the probability of detection [45–47]. To make use of information from these control tags, it is important to develop novel statistical methods that are able to incorporate observational error when the aim is to infer precise positional data from acoustic tracking. Only then can the movement mechanisms that generate a given home range pattern be accurately estimated and the between-individual variability in home range behaviour be properly described.

In this context, state-space models (SSM) have emerged as one of the most promising tools to study animal movement in the wild [40] because they nicely combine a process model (i.e., the movement model that predicts fish position at any time) with an observation model (i.e., the model that properly infers the fish position from the data generated by the tracking system) [48–50]. In SSM, the process model predicts the future fish position given its current position and the mechanistic properties of the movement model, while the observation model provides the probability of obtaining a particular observation (i.e., the number of detections events per receiver per time unit) conditional on the true (and unobserved) fish position [40,51]. The environmental-related changes in the probability of detection can be monitored through control tags located at known positions within the array, and they are included in the modelling process through the observation model [20]. Pedersen and Weng [52] developed an innovative SSM approach that combines a bivariate OU movement model with an appropriate observational model for acoustic tracking data, and demonstrated its robustness and usefulness for estimating the parameters characterizing the home range movement in a coral reef fish.

Here, based on the same conceptual SSM proposed by Pedersen and Weng [52], we present an alternative Bayesian fitting strategy for estimating the parameters of an OU movement model (exploration rate, location and size of the home range). Pedersen and Weng’s SSM solution is based on frequentist statistics, while ours is based on a Bayesian framework. Our methods are not meant to revive the frequentist-Bayesian debates. Instead, our approach should be considered as a convenient alternative for those used to the technicalities and the Bayesian way of reasoning. The Bayesian SSM developed here is highly flexible and it properly deals with the data-sets produced by acoustic tracking arrays. Moreover, it is easily customizable by any other end-user because an R-code is provided associated with our paper (see S1 Appendix). We developed the model framework and tested its robustness via extensive computer simulation before fitting it to a real data-set of a small-bodied coastal fish. The case study presented here is deliberately simple, but it is representative for many small-bodied sedentary coastal species (e.g., those inhabiting reefs or other temperate coastal habitats) in the sense that many of the coastal species have relatively small and stable home ranges [53].

## Materials and Methods

### Ethics Statement

The real data-set is composed of a collection of acoustic detections of wild free-ranging pearly razorfish, *Xyrichtys novacula* tagged with acoustics tags in 2011. The capture and tagging of the individuals were authorized by those responsible for marine natural resources and the Marine Protected Area (MPA) of Palma Bay (Mallorca Island), the Fisheries Department of the Balearic Islands, through a permit to the CONFLICT Project (ref: CGL2008-00958) and to the REC2 Project (ref: CTM2011-23835), both of them funded by the Spanish Ministry of Science and Competiveness. Our study did not involve endangered or protected species, and no animals were sacrificed. Acoustic tags were attached to fish after anesthetization with MS-222, and all efforts were made to minimize fish handling and harm.

### Theoretical assumptions

**Fig 1:**
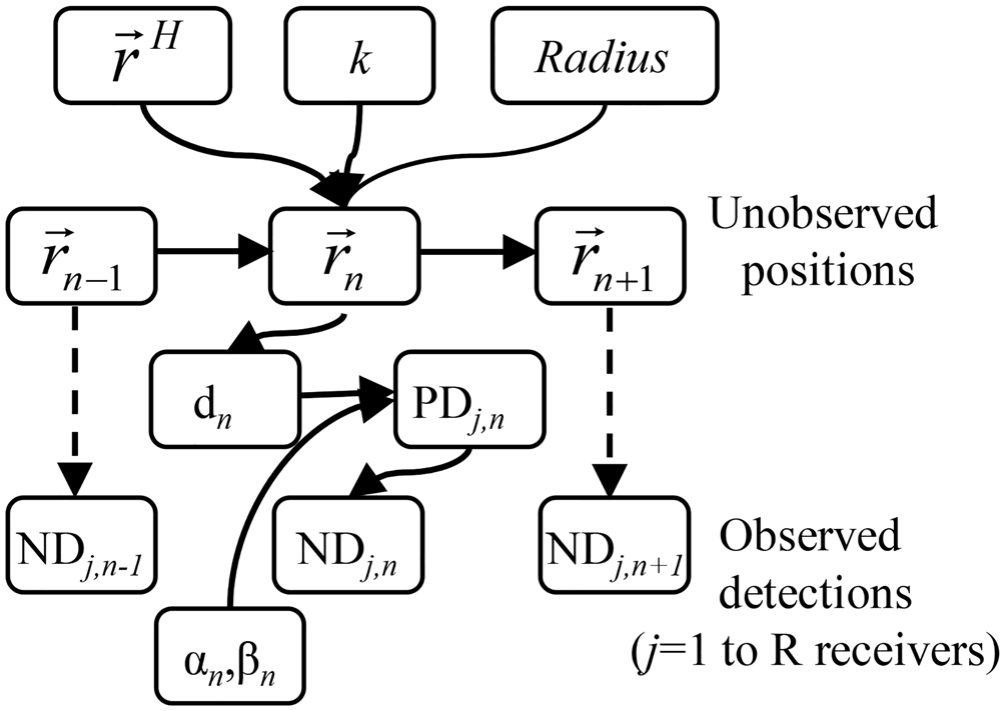
Directed acyclic graph demonstrating the Bayesian state-space model (SSM) approach developed in this paper. The unobserved position $\vec{r}$ at time step *n* is generated following a combination of movement parameters (the process model) of the fish: ${\vec{r}}^{H}$ (position of the center of the home range), *k* and *radius*, and depends in the previous position $\vec{r}$ at *n-1*. The observed data (number of detection, ND) at time step *n* consists in the number of detections over *n* by each of the omnidirectional receivers (*j* in R). Note that ND at *n* is independent of the ND at *n-1* and is generated using the probability of detection by receiver *j* at *n* time unit (*PD*_*j*,*n*_) determined by a logit function (with parameters *α* and *β* at the *n* time) of the distance (*d* at *n*) between the (unobserved) fish position and the (known) receiver position at the *n* time (observational model). The parameters of the state-space model (movement parameters) were estimated using a Bayesian approach.

The SSM developed here assumes that actual fish positions constitute a hidden (unobserved) Markovian state variable that must be estimated from the pattern of detection events on each of the acoustic receivers while following a predetermined mechanistic movement model. Receiver detections of a sound signal emitted by a fish are assumed to constitute stochastic events that depend not only on the distance between the fish and the receiver but also on environmental variables affecting sound propagation [20]. Therefore, our approach combines two different modules: *(i)* the fish movement model, and *(ii)* the observational model (Fig 1).

### Fish movement module: a process model to describe the mechanistic pattern leading to the establishment of a home range

The most widely used model for describing animal movement are random walks (RW) [54,55]. Many different forms of RW have been used to describe the different types of movements encountered in different scenarios and species [8]. The RW case is uncorrelated, i.e., the direction of movement at a given time step is independent of the previous directions of motion, which means that the location at a specific time step depends on the location at the previous time step plus a random term. Moreover, RW assume no bias, i.e., there is no preferred direction of movement. Movement under such circumstances is Brownian, and the pattern produced at the population level is standard diffusion [56]. Simple RWs are, therefore, not a reasonable choice for describing the movement of the increasing number of fish species for which relatively small and temporally stable home ranges have been reported [3]. In these cases, animals do not move freely within large patches of suitable habitat. Instead, there is a need for an additional, possibly memory-driven behavioral rule, according to which each individual will tend to be attached to a specific site [57,58]. Such a movement within relatively small home range can be described by an OU process [8,13]. Accordingly, fish move within a harmonic potential field, the strength of which describes the extent of their home range. The rationale behind this model is that fish still move within a homogeneous environment following random stimuli (e.g., food patches or predatory threat), but this rule is combined with a tendency to remain around a specific point, designated as the center of the home range [14].

Specifically, we consider that the trajectory of a fish, *r(t)* = *(x(t)*, *y(t)*), is described by the stochastic Langevin equation [59]:

$$\vec{r}(t)={\vec{r}}_{H}(t)+\vec{\Delta }(t)$$ (1)

where ${\vec{r}}_{H}$ denotes the position of the center of the home range at time *t*. In general, the center of the home range can be dependent on *t*, expressing for example that during day-time the fish wanders around a particular feeding place, while it is constrained to a different spatial area during night-time.

The displacement $\vec{\Delta }(t)$ from the instantaneous center of the home range at any time is given by the OU process

$$\frac{d\vec{\Delta }(t)}{dt}=-k(t)\vec{\Delta }(t)+\vec{\xi }(t)$$ (2)

which represents a fish that is attracted toward the center of its home range by a central harmonic force of instantaneous strength *k(t)*, while it is also subjected to an external random force. The random force is described by the Langevin term $\vec{\xi }(t)$, which is a bi-dimensional, white Gaussian process of zero mean, variance (*ε*) in each spatial coordinate and no correlation among them ([14], but see [52] for an alternative definition that may translate in elliptical home range). Again, the time dependence of *k* and *ε* expresses that the fish behavior may change across *t*, for instance some species exhibit two different states (e.g., foraging or resting type of movement).

The general solution of Eq 2 is:

$$\vec{r}(t)={\vec{r}}_{H}(t)+e^{-Q(t)}\left[{\vec{\Delta }}_{0}+\int_{0}^{t}\xi (t\prime )e^{Q(t\prime )}dt\prime \right]$$ (3)

where *Q(t)* is given by:

$$Q(t)=\int_{0}^{t}k(t\prime )dt\prime$$ (4)

A suitable discretization (*t* = *nΔt*) of the fish trajectory described by Eqs 3 and 4 is given by:

$${\vec{r}}_{n+1}={\vec{r}}_{n+1}^{H}+e^{-(Q_{n+1}-Q_{n})}\left({\vec{r}}_{n}-{\vec{r}}_{n}^{H}\right)+{\vec{R}}_{n}$$ (5)

where ${\vec{r}}_{n}^{H}$ denotes the position of the center of the home range at time *t* = *nΔt*,

$$Q_{n+1}-Q_{n}=\int_{n\Delta t}^{(n+1)\Delta t}k(t\prime )dt\prime$$ (6)

and ${\vec{R}}_{n}$ is a stochastic, normally distributed, variable with zero mean and standard deviation (*σ*):

σn=εn(1−e−2knΔt)2kn (7)

Eqs 1 to 7 apply to the general case in which ${\vec{r}}^{H}$, *k* and *ε* may be time-dependent and define different behavioral states. However, here we develop the simplest case applied to species with diurnal active life-styles. For example, our case species, the pearly razorfish remains inactive and buried in the soft bottom during night-time (see below for more details of the biological model selected for the real data-set). When the parameters of the movement model are constant (e.g., during day-time in the pearly razorfish), Eqs 1–7 simplify, and the movement of the fish can be described by

$${\vec{r}}_{n+1}={\vec{r}}_{}^{H}+e^{-k\Delta t}\left({\vec{r}}_{n}-{\vec{r}}_{}^{H}\right)+{\vec{R}}_{n}$$ (8)

where ${\vec{R}}_{n}$ is a stochastic, normally distributed, variable with zero mean and standard deviation (*σ*):

σ=ε(1−e−2kΔt)2k (9)

The biologically relevant effect is that the movement of the fish is stochastic within a given spatial area surrounding the center of the home range. The “radius” of the circular home range (*radius*, the radius of the area within which a fish has a 95% probability of being found when a large period of time is considered) depends on *k* and *ε* [14]:

$$radius_{}=\sqrt{\frac{-\varepsilon_{}\text{ ln}\left(1-0.95\right)}{k_{}}}$$ (10)

Palmer et al. [14] developed the biological interpretation of this specific version of a random walk for marine coastal fishes. While the size of the circular home range (*radius*) depends on the ratio *ε*/*k* and determines the potential size of the space use of the individual in meters, the parameter *k* of the model is the rate of exploration (in min^-1^), which determines the slope of the curve describing the cumulative space used in function of time or how quickly the individual explores the whole home range. Thus, *k* represents the speed by which an individual moves through its home range.

### Observational module: modelling the probability of detection using control tags

The second module of our SSM deals with the observational model (Fig 1). As commented above, the true fish positions $\vec{r}(t)$ are unobserved. Instead, the only information obtained by an array of acoustic receivers consists of a detection pattern (i.e., how many detections are registered during the *n* time-steps by each of the acoustic receivers of the listening array, or *ND*_*n*,*j*_). The probability of detecting a signal (*PD*_*n*,*j*_) is described by a logistic function [20,42,44,52] of the distance $d_{n,j}=\sqrt{{\left(x_{n}-x_{RECj}\right)}^{2}+{\left(y_{n}-y_{RECj}\right)}^{2}}$ between the true fish position at *n*, (*x*_*n*_, *y*_*n*_), and receiver *j* (*j* in R receivers), located at (*x*_*RECj*_, *y*_*RECj*_),

Log(PDj,n1−PDj,n)=α+β[(xn−xRECj)2+(yn−yRECj)2] (11)

### Parameter estimation

Given the input data (a matrix consisting in the number of detections, *ND*_*n*,*j*_, at each one of the *j* receivers during *n* time-steps), the goal is to estimate both the value and uncertainty of the movement parameters (${\vec{r}}^{H}$, *k* and *radius*). In the fish movement module (upper level in Fig 1), we used the movement model defined by Eqs 8 and 9, and for the observation module (lower level in Fig 1), we used Eq 11. Concerning such an observation module, it is well known that a detection event mainly depends on the distance between a fish and a receiver (*α* and *β* in Eq 11) but it is also influenced by environmental conditions [42–44]. These dependencies are explicitly modelled and estimated from the input data, and *α* and *β* were estimated in our application using a control tag moored at known distances from each of the receivers [47]. The temporal scale at which *α* and *β* should be estimated is case-specific. In our case, with no tidal variations, a daily scale was chosen for simplicity (see below). This means that the values *α*_*day*_ and *β*_*day*_ were considered constant at the within-day scale. Again, for simplicity, *α*_*day*_ and *β*_*day*_ were estimated in preliminary and independent statistical analyses, and were considered fixed and supplied as data to the Bayesian model detailed below.

The movement parameters (and uncertainty) of the SSM were estimated using a Bayesian fitting strategy [60]. The model was implemented and run using the R2jags library of the R package (http://www.r-project.org/), which opens JAGS (http://mathstat.helsinki.fi/openbugs/). Three Markov Chains Monte Carlo simulations (MCMC) were run, and minimally informative prior knowledge was assumed (see S1 Appendix). Specifically, *k* was assumed to follow a uniform distribution bounded between zero and 1 min^-1^. The *radius*, and the latitude and the longitude of the center of the home range were assumed to follow a normal distribution. In all four cases, the parameters of the prior distributions ensured a nearly flat prior distribution (see S1 Appendix). In addition, for demonstrative purposes, we compared the posterior distributions resulting when minimally informative priors were used with those obtained when biological information is available for setting the priors, and when alternative prior distributions were imposed. The first 10,000 iterations for all of the parameters were discarded (burn in period), and a thinning strategy was adopted to ensure the temporal independence of successive values within the chain (only one out of every 10 consecutive values was kept). The convergence of the MCMC chains of all parameters was assessed by visual inspection of the plots of the iterations and tested using the Gelman-Rubin Statistic [61], with values < 1.1 indicating convergence [62]. Convergence was reached after a variable number of iterations. Depending on the simulation or the real case of tagged fish (see below), between 3,000 and 9,000 valid iterations were retained after burning and thinning for describing posterior distributions. A fully customizable R-code (corresponding to one simulation experiment; see below) is provided in S1 Appendix.

### Precision and accuracy of the analytical approach: simulation experiments

Before applying the Bayesian SSM described here to a real data-set, the accuracy and precision of the estimations and the effect of the prior distribution in the posteriors were checked via computer simulation. The simulation experiments were aimed at disentangling the effects of two issues when estimating the movement parameters: *(i)* different combinations of movement parameters in which the exploration rate (*k*) and the *radius* of the home range varied mirroring the between-fish variability observed in the real study-case (sim 1 to 4, Table 1), and *(ii)* the effect of the observational time-step, defined as the fraction of time where the detections are pooled (5, 10, 15, 30, 60 and 90 min). Note that in all the simulation experiments, the transmitter emitted one acoustic signal per minute, which is the actual emission period in the case of the pearly razorfish (PT-2, Sonotronics, Inc., Tucson, Arizona, USA; [63]). Therefore, the simulated fish was moved every minute and the detection (or not) by each of the receivers in the array was checked with the same periodicity. However, depending on the specific simulation experiment, the detections were pooled in different time-steps as mentioned above and would be typical in real applications.

**Table 1:** Characteristics of the temporal series of acoustic detections considering four combinations of movement parameters (sim 1, sim 2, sim 3 and sim 4) to test the performance and accuracy of the Bayesian state-space model developed here. The table shows the number of days of the time series and the total number, mean and s.d. of detections generated considering a time-step period of 15 min. The table also shows the specific values of the movement parameters simulated (exploration rate of the home range *k* min^-1^, the *radius* in meters of the circular home range, and the latitude and longitude in meters of the center of the home range).

	Characteristics of the simulated temporal series of detections					Movement Parameters (simulated)			
Simulation ID	Days	Initial day	Detections	Mean	s.d.	*k* (min^-1^)	*Radius* (m)	Longitude (m)	Latitude (m)
Sim 1	12	2011-08-01	31671	47.1	4.9	0.001	245	0	0
Sim 2	12	2011-08-01	30617	45.6	5.0	0.001	387	0	0
Sim 3	12	2011-08-01	31343	46.6	4.8	0.01	245	0	0
Sim 4	12	2011-08-01	31466	46.8	4.8	0.01	387	0	0

**Fig 2:**
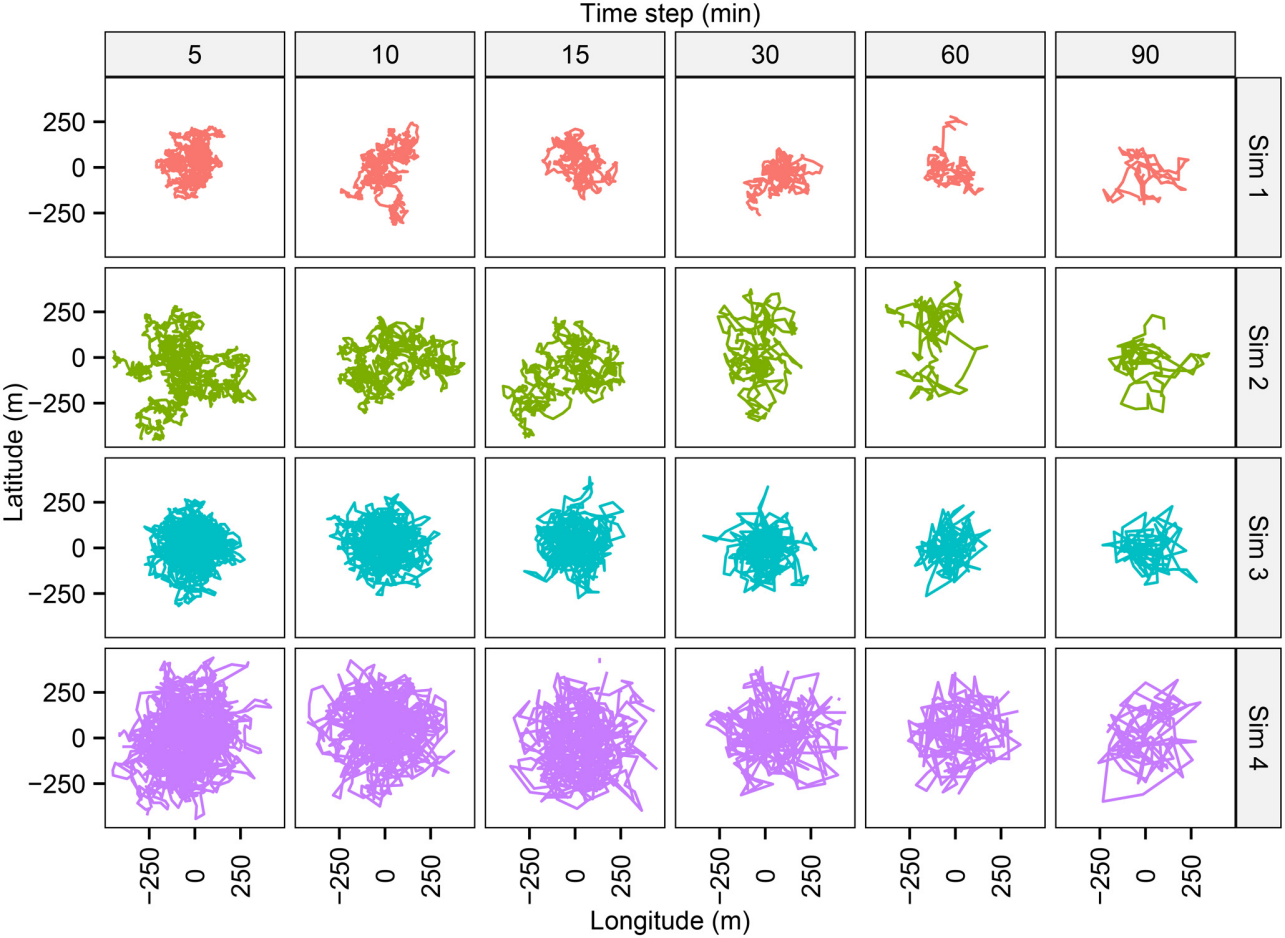
Simulated data for testing the feasibility and accuracy of our approach. Discrete-time trajectories of the four movement parameters combinations (sim 1, sim 2, sim 3 and sim 4, Table 1) generated for 6 different time-steps periods in min (5, 10, 15, 30, 60 and 90 min) to test the accuracy and the performance of the analytical approach. The resulting numbers of acoustic detections were obtained through the simulation experiment for the 24 movement trajectories, and the movement parameters and positions were estimated using a Bayesian tate-space model proposed here. Results were compared with the (known) real values.

**Fig 3:**
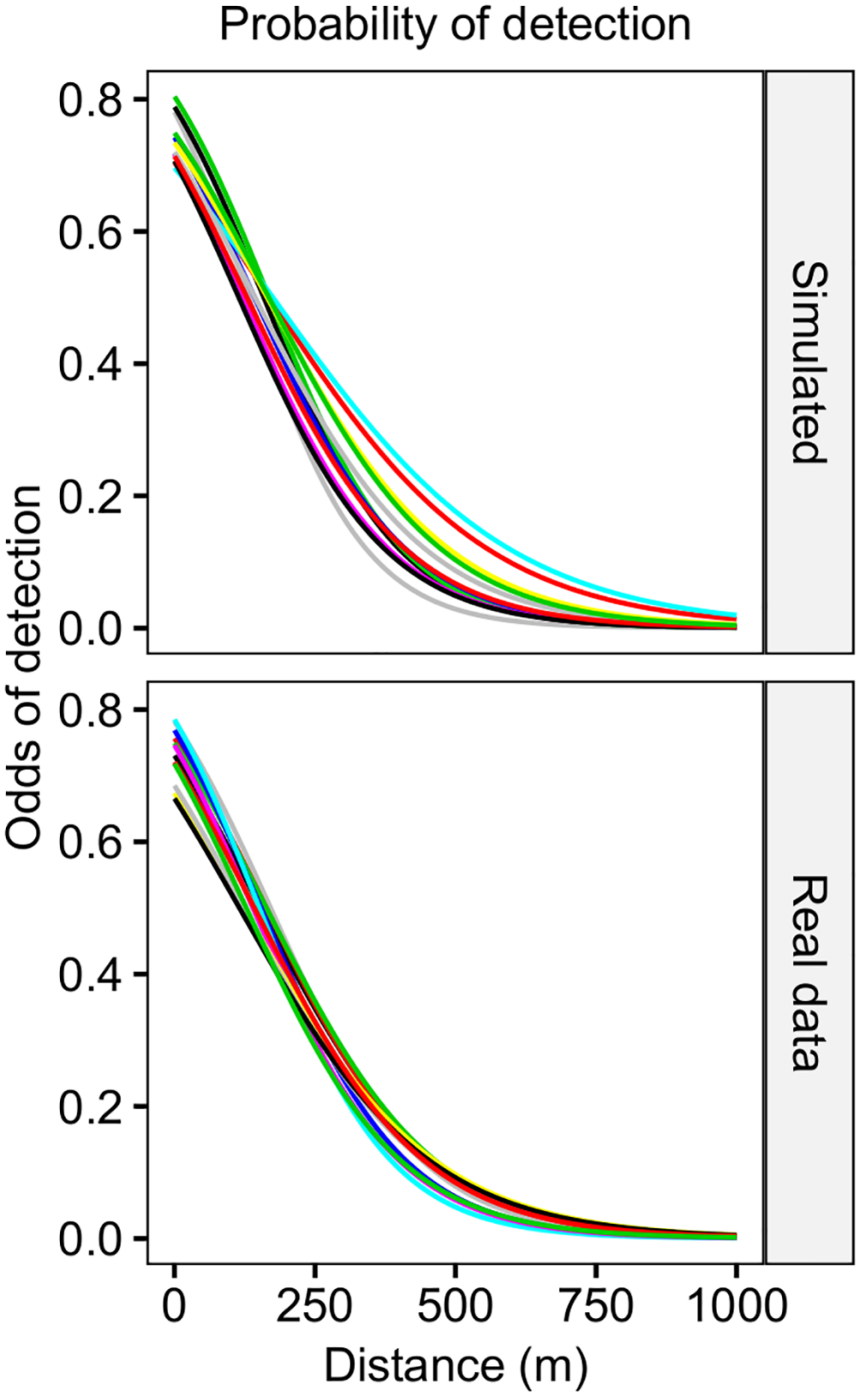
Simulated and real probability of detection in function of the distance. Daily (*n* = 10 days) probability of detections (logit function with parameters *α* and *β*) against the distance considered in the simulated exercise (upper panel) and real-data (down panel) implemented in the observational model of the Bayesian state-space model described in Fig 1. Note the low variability observed in our case study. The variability in the logit models can be easily modified and adapted to any other case as the parameters of the model (*α* and *β*) can be estimated (using a control tag) and included in the analytical approach for each time step (see Material and Methods).

Two series of simulations were conducted. In the first, we generated a total of 24 fish trajectories using Eqs 8 and 9 (Fig 2) considering four realistic combinations of movement parameters (*k* and *radius*; Table 1), which were analysed after pooling the number of detections at the 6 different observational time-steps. A simulated squared array of 25 evenly spaced (300 m) omnidirectional receivers and a sequence of *α*_*day*_ and *β*_*day*_, estimated using control tags, both inspired by the settings of the acoustic tracking study described by Alós et al. [63], were used to generate a matrix of acoustic detections per time-step (ND_*n*,*j*_) for each one of the 24 simulated trajectories (see S1 Appendix). The tracking period was 12 days. However, to mirror diel behavior of the razor fish, the fish was moved only during 14 light-hours per day, which means that a fish path lasted for 10,080 positions (or 12×14×60 min). Therefore, the data input for the Bayesian analyses of each simulation experiment consisted of a multivariate temporal series of number of detections per time-step, which was a matrix of 25 columns (receivers) by 2016, 1008, 672, 336 or 168 rows (for a time-step of, respectively, 5, 10, 15, 30, 60 and 90 minutes). Concerning the detection probability, we generated a time sequence of *α*_day_ and *β*_day_ based on the between-day variability currently observed, which has been assessed by fitting the number of detections actually obtained by control tags moored in a known position in the array of acoustic receivers [63]. In our case, the choice of a daily scale for the detection probability is justified by the low temporal variability observed in the real case study (Fig 3) [63]; this scale should be modified to fit the case specificities of future acoustic tracking studies (see S1 Appendix).

In summary, for each of the 24 simulations, fish were moved within the array and a new position was defined after one minute (Eqs 8 and 9). The probability of detection by each one of 25 omnidirectional receivers was then calculated as a function of *(i)* the distance between the fish and the receivers and *(ii)* the day-specific values of *α*_*day*_ and *β*_*day*_ (Eq 11). These predicted probability values were compared with random values extracted from a uniform distribution between zero and one to simulate detection (or not). Finally, the number of detections by each receiver was cumulated according to the specific time-step. We then fitted the Bayesian SSM to the input data produced by these 24 simulation experiments and the estimated movement parameters (posterior median and Bayesian Credibility Intervals, BCI, 2.5% and 97.5% for *k*, *radius* and the position of the center of the home range) were compared with the true (known) values.

The second series of simulations aimed assessing the accuracy and the precision of the Bayesian model, in particular the effect of the priors in the posterior distributions. These second series of simulations were focused in the most extreme scenarios in terms of movement parameters (sim 1 and sim 4, Table 1). The goal was to obtain precise estimates of accuracy and precision considering a time-step of 15 min and 30 min according to the first block of simulations (see result below for relevant bias starting at time-steps of 30 min). Three simulations scenarios were then considered: *(i)* sim 1 with a 30 minutes time-step, *(ii)* sim 4 with a 30 minutes time-step and *(iii)* sim 4 with a 15 minutes time-step in line with the results of the first set of simulations. In these three cases, instead of a single fish, the simulation experiment was repeated for 50 fish to obtain 50 replicates of each simulation. The percentages of simulations where the known parameters were properly estimated (where the estimated BCI of the parameter included the true value) were quantified. Finally, the outcomes of imposing different priors were evaluated using a single simulation by comparing the posterior distribution and a set of five different prior distributions. We focused only on the case of the *radius* of the home range because data were more easily available for this parameter. In the case of the pearly razorfish, the 95% of the kernel utilization distribution occurred within an averaged (between-fish) accumulated area and s.d. of 0.32 ± 0.13 km^2^ which is equivalent to a radius of 314 ± 67 m [63]. By providing a fish with a true radius of 245 m (sim 1 above), the BCI were compared after setting five different priors.

### Case study—the pearly razorfish, *Xyrichtys novacula*

The Bayesian SSM was applied to a collection of acoustic detections from an acoustic tracking experiment done in 2011 where the movement of several pearly razorfish was monitored for a short period of time (~20 d: the length of tracking period is limited by the battery life span, which in turn is limited by the fish size) using an array of 21 omnidirectional acoustic receivers (model SUR-1, Sonotronics, Inc., Tucson, Arizona, USA) in the waters of Mallorca Island, NW Mediterranean (see the details of the receivers array in [63] and S1 Dataset). The pearly razorfish is a small protogynous monandric hermaphrodite with marked sexual dimorphism [64,65] and prefers habitats characterized by sandy soft bottoms [66,67]; the species is highly targeted by the recreational fisheries in temperate areas in the Mediterranean [68].

We selected six tracked individuals in 2011 that generated sufficient data following the decision-tree criteria to discard potential mortalities described in March et al. [69]. Moreover, it is well known that after the implementation of the acoustic tag *X*. *novacula* show a short period of abnormal behavior during which the fish remain buried in the soft [63]. Accordingly, we used Continuous Wavelet Transformations (CWT) using the *sowas* library in R-package to detect the normal behavior to set the initial day of the time-series of acoustic detections following Alós et al. [63]. We only considered the day-time detections as the pearly razorfish remains inactive and buried in the soft bottom during the night-time, which prevents detections [63]. The resulting time series of detections for each tagged fish are presented in Table 2 and are representative of the typical data generated in this type of acoustic tracking study based on arrays of omnidirectional receivers (e.g., [70–72]). We fitted the Bayesian SSM to these 6 tagged fish, and the posterior distribution of the movement parameters (latitude and longitude of the center of the home range (${\vec{r}}^{H}$), *radius* and *k*) and their uncertainty (BCI) were summarized for each individual. Following the results of the simulation exercise (see Results), we decided to fit the Bayesian SSM considering a time-step of 15 min.

**Table 2:** Characteristics of the time-series of acoustic detections obtained from 6 individual of pearly razorfish, *Xyrichtys novacula* tracked in the waters of Mallorca Island (NW Mediterranean). The table shows the characteristics of the individuals tracked, the acoustic tag identification (ID) and the gender and the total length in mm of the individual. The time-steps defined the number of 15 min periods (*n*), the total detections shows the overall number of detection received by a given individual, and mean and standard deviation (s.d.) of detections shows the average number of detection per time-step generated for each individual tracked.

Fish ID	Gender	Total length	Time-steps (*n*)	Days	Initial Day	Total Detections	Mean detections	s.d. detections
201102	Male	187	842	15	01/08/2011	27709	33.1	18.2
201104	Male	209	842	15	01/08/2011	36885	44.1	20.4
201107	Male	185	842	15	01/08/2011	21123	25.3	17.0
201109	Female	158	675	12	01/08/2011	9158	9.8	8.6
201111	Female	159	842	15	01/08/2011	39468	47.2	12.1
201113	Male	192	842	15	01/08/2011	48940	58.4	19.8

## Results

### Simulation experiments

**Fig 4:**
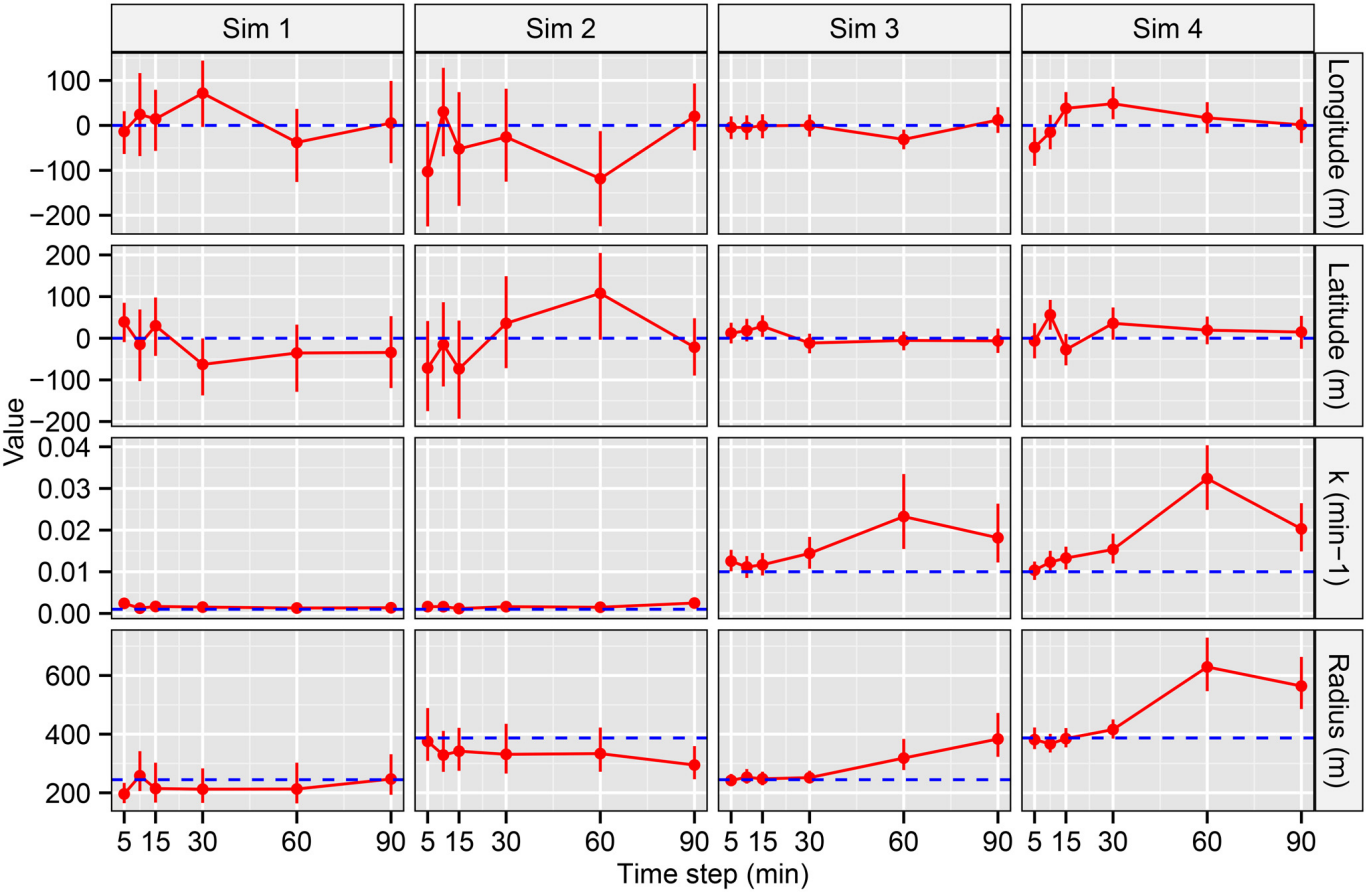
Estimated and real movement parameters resulted from the simulation experiment. Estimated Bayesian Credibility Intervals (BCI, 2.5% and 97.5% as point range in red) using the Bayesian state-space model proposed here and real values (as a horizontal dashed blue line) of the movement parameters (latitude and longitude in meters of the center of the home range, *k* in min^-1^ and *radius* in meters) in each of the combinations of different time-steps considered here (5, 10 15, 30, 60 and 90 min) and four simulations (overall 24 simulated trajectories). In most cases the estimated BCI included the real value suggesting good performance of the model. Only the estimated values for the time-steps periods 30, 60 and 90 min in simulation 3 and 4 were consistently biased, suggesting a poor performance for this particular type of fish movement.

The Bayesian SSM retrieved the movement parameters of the simulated fish trajectories with acceptable precision and accuracy in most of the combinations of movement parameters (sim 1, 2, 3 and 4) and for the six time-steps that were considered (Fig 4). The Bayesian Credibility Intervals (BCI, 2.5% and 97.5%) were usually tight, and in most cases (84.4%) the true value was within the BCI, which indicated that the results obtained with the Bayesian approach were accurate in most of the cases (Fig 4). Regarding the estimation of the latitude and longitude of center of the home range (${\vec{r}}^{H}$ in meters), the model fit yielded BCIs that included the real value in almost all cases (Fig 4). However, the uncertainties associated with estimating ${\vec{r}}^{H}$ were larger in sim 1 (*k* = 0.001 min^-1^ and *radius* = 245 m) and sim 2 (*k* = 0.001 min^-1^ and *radius* = 387 m) than in sim 3 (*k* = 0.01 min^-1^ and *radius* = 245 m) and sim 4 (*k* = 0.01 min^-1^ and *radius* = 387 m). Regarding the exploration rate (*k* in min^-1^), the Bayesian SSM retrieved BCIs that included the true value except for the two largest time-steps (i.e., 30, 60 and 90 min), where *k* was overestimated (Fig 4). Moreover, precision of *k* was smaller in sim 3 and 4, suggesting positive relationship between uncertainty and how fast the individual explores the home range (Fig 4).

**Fig 5:**
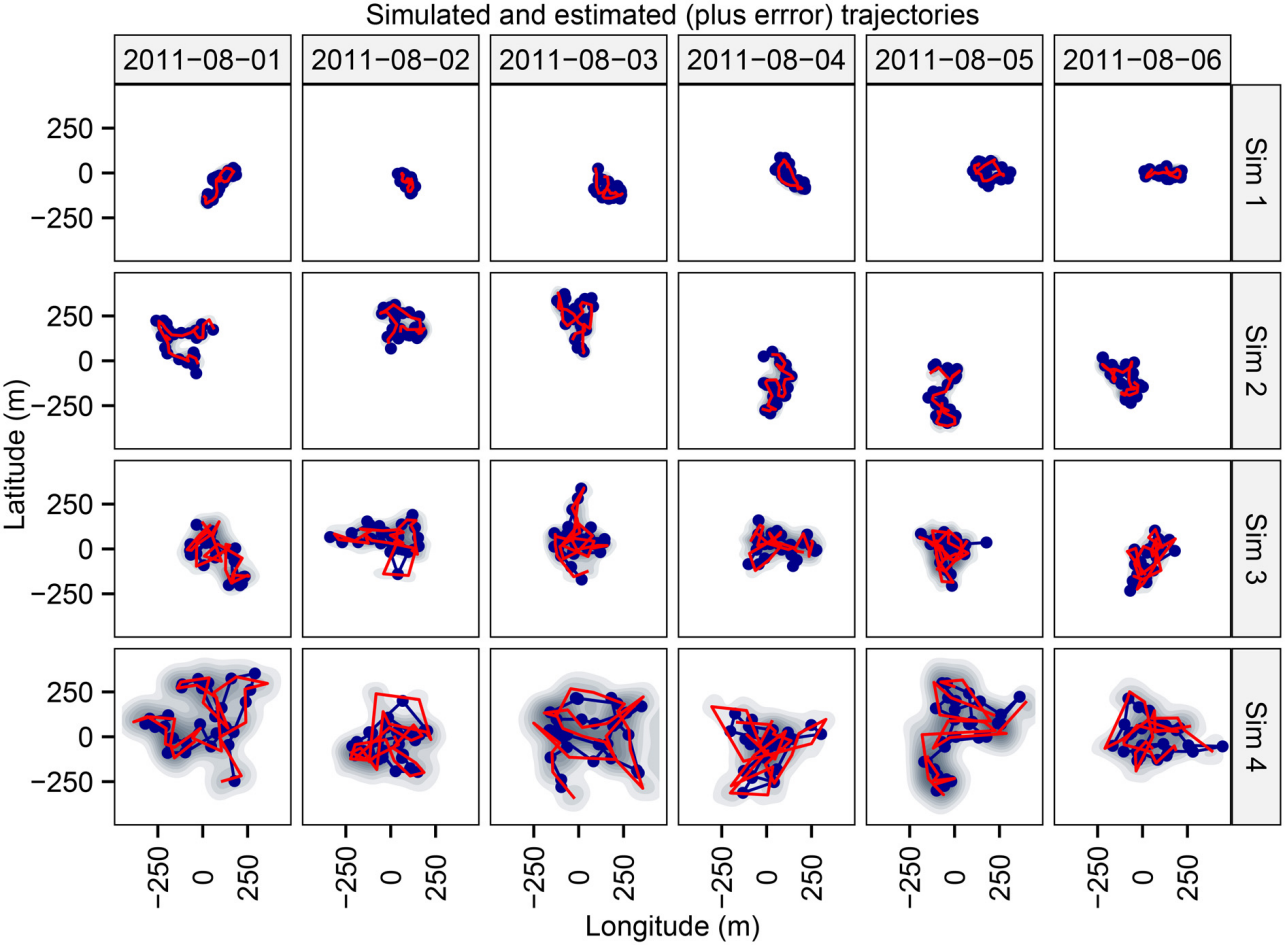
Estimated and real fish trajectories resulted from the simulation experiment. First six days of the estimated (in blue) and real (in red) discrete-time trajectories of the 24 simulated trajectories. The estimated trajectory corresponds to the Bayesian mean, and the error is represented in the figure as a density plot of 100 trajectories re-sampled from the posterior distribution generated by the state-space model.

The estimations regarding the *radius* were similar to the results obtained for *k*: most of the estimated BCIs included the true value, with the exception of the two largest times-steps (60 and 90 min) where the *radius* was overestimated (Fig 4). In the case of the *radius*, the uncertainties associated with the estimation of the parameter were similar for all combinations (Fig 4). Overall this first series of 24 simulation experiments suggested a good performance and accuracy of the Bayesian SSM unless the time-step is large (30, 60 or 90 min), especially for the simulations involving large exploration per unit of time (i.e., high *k* in simulation runs 3 and 4, Table 1). Moreover, the Bayesian SMM also seems to properly retrieve the original trajectory of the simulated fish, which supported a good performance for positioning the fish (Fig 5).

The results obtained from the second series of simulation experiments (50 replicates) confirmed the general picture depicted in the first series of simulation experiments for a single fish (Table 3). When *k* and *radius* were small (sim 1), the largest time-step considered (30 min) had no or small effect, and the estimates of the movement parameters were accurate, which suggested a good performance of the Bayesian SSM (Table 3). However, when *k* and radius were larger (sim 4), the largest time-step produced slightly biased (overestimation) estimates for *k*, while the other parameters remained unbiased (Table 3). The overestimation observed in *k* was notably reduced when smaller time-steps (15 min) were used, and the percentage of BCI containing the true value raised from 8% to 78% suggesting a better performance of a 15 min time-step (or smaller) than 30 min (or larger) when *k* and radius were large (Table 3).

**Table 3:** Percentages of agreement (% of replicas where the estimated Bayesian Credibility Interval, BCI, 2.5% and 97.5% included the true-known value) for each movement parameter obtained from the second series of simulation experiments based in 50 replications of sim 1 and sim 4 considering a time-step of 15 and 30 min.

	(%, *n* = 50) Time-step = 15 min			
Simulation ID	*k*	*Radius*	Longitude	Latitude
Sim 1	96%	92%	98%	98%
Sim 4	78%	90%	92%	90%
	(%, *n* = 50) Time-step = 30 min			
	*k*	*Radius*	Longitude	Latitude
Sim 1	98%	92%	98%	100%
Sim 4	8%	86%	90%	90%

The outcomes of imposing different priors were evaluated using a single simulation. For a uniform distribution bounded between 0 and 10,000 m, the estimated BCI of the *radius* (real value 245 m) was 185 and 405 m. For a normal distribution with zero mean and a large variance (tolerance = 10^−8^), the BCI was 178 and 417 m, and for a normal distribution with mean = 314 m and the observed between-fish variance, the BCI was 198 and 357 m. Finally, for a normal distribution with mean = 314 m and a variance ten times larger than the observed between-fish variance, BCI was 193 and 357 m. Therefore, when minimally informative or reasonable priors were assumed, posteriors were largely narrower than priors, and BCIs were similar and included the true value. Conversely, for a normal distribution with mean = 100 m and a narrow variance (tolerance = 0.01), BCI was 133 and 157 m. Therefore, as expected, when very informative, but biased priors were assumed, posterior distributions did not include the true value.

### Case study of pearly razorfish

**Fig 6:**
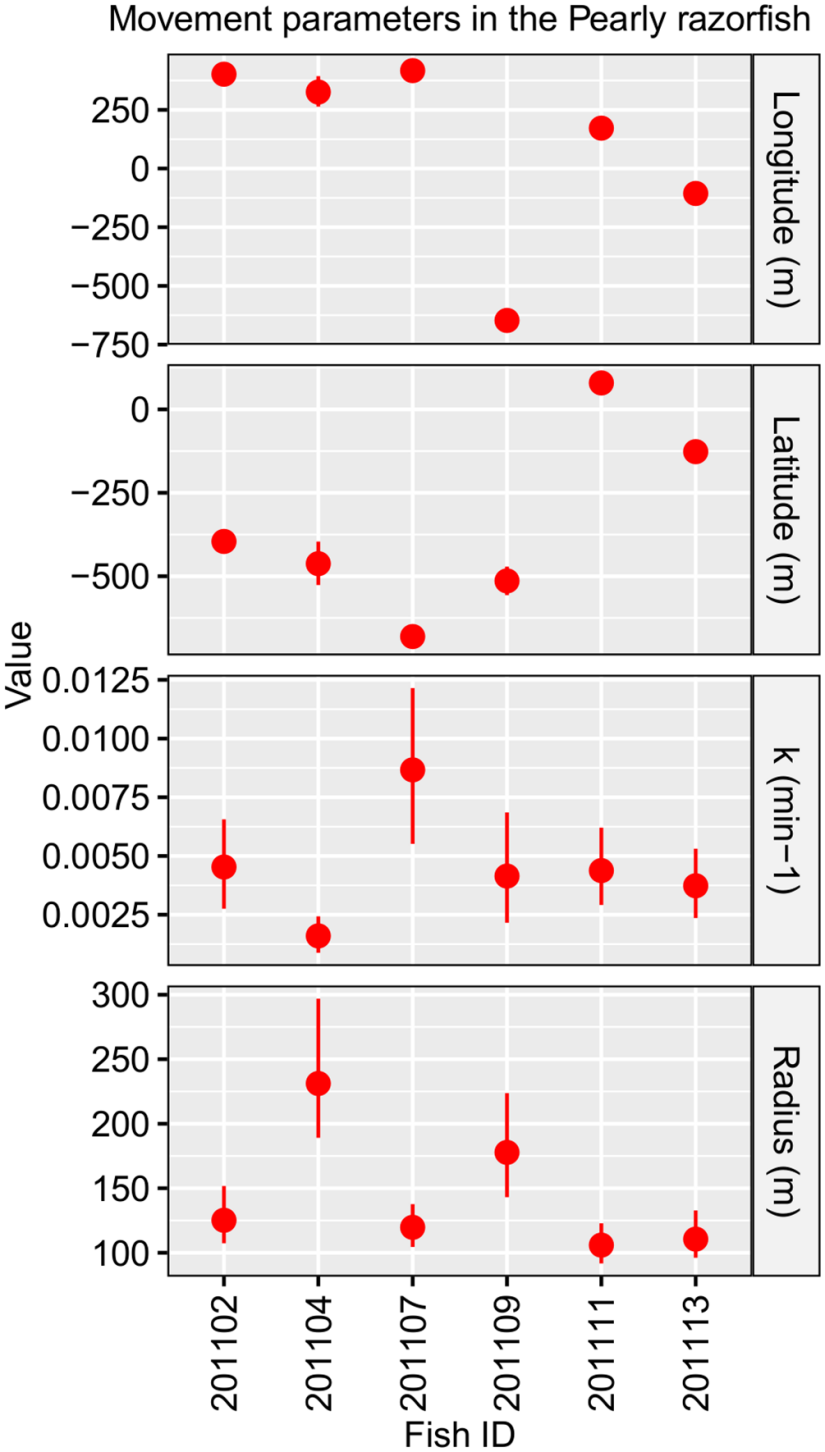
Estimated (plus uncertainty) movement parameters in case study applied to pearly reazorfish *Xyrichtys novacula*, using a Bayesian state-space model. Estimated Bayesian Credibility Interval (BCI, 2.5% and 97.5% as point range in red) of the movement parameters using a Bayesian state-space model for discrete-time (based in a time-step of 15 min) trajectories of 6 individuals of pearly razorfish, *Xyrichtys novacula* tracked in 2011 using an array of omnidirectional receivers in the waters of Mallorca Island (NW Mediterranean).

**Fig 7:**
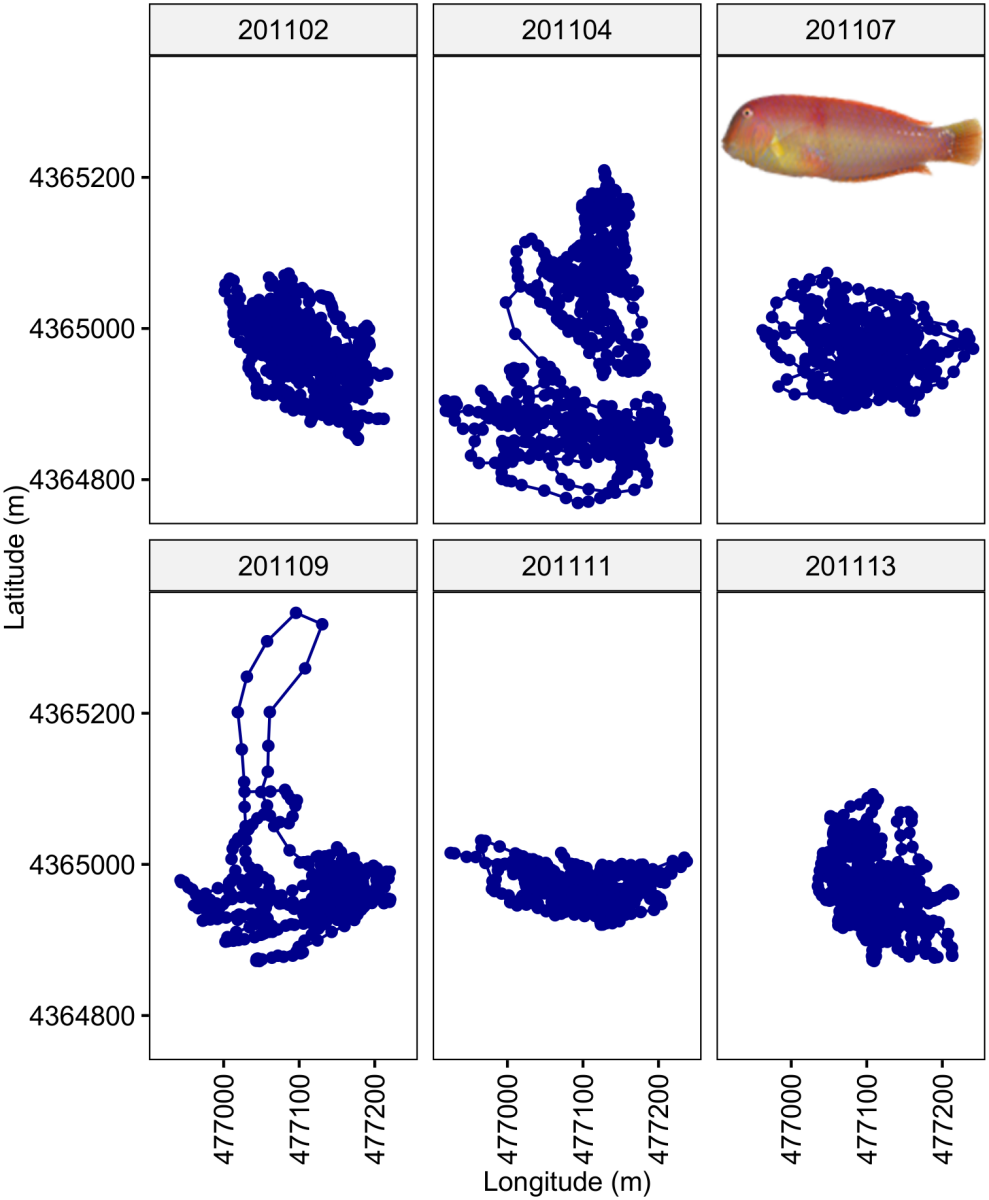
Estimated trajectories in case study of pearly razorfish, *Xyrichtys novacula*, using a Bayesian state-space model. Estimated trajectory using a Bayesian states-space model for discrete-time (based in a time-step of 15 min) trajectories of six individuals of pearly razorfish, *Xyrichtys novacula* (the picture shows an image of the species) tracked in 2011 using an array of omnidirectional receivers in the waters of Mallorca Island (NW Mediterranean). The plot shows the continuous path and the estimated positions as points in latitude and longitude (UTM). The trajectories have been centered to the same center of the home range to improve visualization.

The results of the simulation experiments above suggested that a time-step of 15 minutes or less provided the most accurate and precise estimates of the movement parameters in most of the simulation cases that were evaluated. As computation time exponentially increases with the size of the input data, we considered a time-step of 15 min for fitting the real data set (~ 3 h per individual). The Gelban-Rubin statistic, which assessed the convergence of the model parameters, was below of 1.1 in all cases. Table 4 shows the BCIs of the estimated movement parameters for the 6 individuals of pearly razorfish that were analysed. The BCIs did not overlap in many cases, suggesting the existence of individual differences in the movement parameters (Fig 6). Fig 7 displays the estimated trajectories of the individuals tracked showing different patterns of home range behavior.

**Table 4:** Posterior distributions of the Bayesian state-space model fitted to the temporal series of acoustic detection generated by 6 individuals of pearly razorfish, *Xyrichtys novacula*, tracked in 2011 to estimate the home range movement parameters (the exploration rate of the home range *k*, in min^-1^), the *radius* of the circular home range (in m) and the latitude and the longitude in UTM). The table shows the Bayesian mean and the uncertainty associated with the movement parameters through the Bayesian Credibility Interval (BCI, 2.5% and 97.5%).

	Movement parameters (real)											
	Longitude (m)			Latitude (m)			*k* (min^-1^)			*Radius* (m)		
Fish ID	Mean	BCI 2.5%	BCI 97.5%	Mean	BCI 2.5%	BCI 97.5%	Mean	BCI 2.5%	BCI 97.5%	Mean	BCI 2.5%	BCI 97.5%
201102	477412.9	477390.8	477433.2	4364924.2	4364901.5	4364945.2	0.005	0.003	0.007	125.2	107.4	151.7
201104	477337.0	477274.6	477404.7	4364857.7	4364793.8	4364923.9	0.002	0.001	0.002	231.2	189.1	296.9
201107	477428.1	477412.8	477444.4	4364639.2	4364622.3	4364656.0	0.009	0.006	0.012	119.7	104.5	137.7
201109	476363.7	476316.4	476410.7	4364806.0	4364763.3	4364848.6	0.004	0.002	0.007	177.8	143.1	223.7
201111	477183.0	477166.0	477200.3	4365399.4	4365382.3	4365415.8	0.004	0.003	0.006	105.9	91.7	122.8
201113	476904.8	476885.6	476922.6	4365193.3	4365176.4	4365213.3	0.004	0.002	0.005	110.5	96.1	132.8

## Discussion

A particular movement behavior that constraints the animal within a small area or home range has several ecological, evolutionary and managerial consequences for many exploited fish species. With the recent miniaturization of acoustic tracking devices, fisheries scientists and ecologists have now a suitable tool for disentangling the mechanisms behind the behavior that constraints fish within a home range or, more generally, behind any movement behavior [27,28], even for small-bodied fish such as the pearly razorfish. However, given that the data collected by arrays of omnidirectional acoustic receivers (i.e., number of detections per time unit) are only indirectly related to the fish’s position, the estimation of the movement parameters is often imprecise. This may in turn limit the biological interpretation of acoustic tracking data [40]. In this paper, we presented a Bayesian approach for fitting a SSM, which joins a mechanistic home range movement model (in our case, a random walk weighted by an OU process), with an appropriate observational model. This observational model predicts detection probabilities not only from the distance between a fish and the receivers, but it also takes into account that the distance-related detectability can be affected by environmental factors. Related work combining movement and observational models based on a frequentist solution for estimating the movement parameters in a SSM have recently been published [52]. The Bayesian solutions we propose follows this research and constitute a suitable alternative for those used to the Bayesian way of reasoning. With both a frequentist [50] and a Bayesian SSM approach available, the methodological ground is developed for improving the mechanistic understanding of home range behavior and its ecological and evolutionary consequences across a wide range of animals.

SSMs are among the most promising analytical tools for analyzing movement data generated from tracking systems [40,49], including acoustic applications [51,52]. All SSM are based on combining two models: *(i)* the process and *(ii)* the observational model. Concerning the process model, the specific movement model we choose for describing the movement behavior of our study species is able to unravel the behavioral mechanisms behind the emergence of a spatially confined home range, not only in terms of its size (*radius*), but also the location of the center of the home range, and to study how quickly a fish explores its home range (parameter *k*, [14]). These parameters can be interpreted as individual traits that might be under selection by natural or anthropogenic forces [77]. Moreover, the SSM modelling approach is flexible and it can easily accommodate other types of movement behaviors if they fit the specificities of the tracked species (e.g., correlated random walks following an environmental driver, [73]). One of the key improvements of SSM is the incorporation of an observational error module. In the same way that is demonstrated in [52], our approach allows for the explicit incorporation of the effects of environmental variability over the general pattern of a distance-dependent detection probability (Fig 1). Specifically, the detection probability of an acoustic pulse transmitted by a fish was described by a distance-dependent logistic model, shown in Eq 11 and elsewhere [42,52,74]. The parameters of the logistic curve (*α* and *β*) can be estimated at the desired temporal scale using a control tag (as in our case), thereby providing a solution for addressing the variability in the probability of detection related to environmental factors [20].

One of the main novelties of the approach proposed here is technical rather than conceptual, in the sense that the model parameters are estimated using the Bayesian machinery. Bayesian inference has been widely proposed as an efficient way to estimate the parameters of complex movement models with large number of parameters [49], as is the case in the SSM proposed here. Another potential advantage is that the Bayesian inference method allows combining existing knowledge (through prior probabilities) with additional knowledge derived from new data (through likelihood) to obtain the posterior distribution of the model parameters (in our case the movement parameters and movement path) [75]. The posterior distribution of the MCMC summarizes the degree of belief of the parameter estimates, given the data. However, rather than promoting frequentist-Bayesian debates, which is not intended by our work, the results we obtained strongly support that posterior distributions and its biological interpretation are virtually the same when imposing either minimally informative priors or reasonable informative priors. Conversely, only when very informative yet possibly biased priors are imposed, the analysis may result in biased posteriors as demonstrated in our simulation analysis. However, our simulation experiments have also demonstrated that the accuracy and precision in the estimation of the movement parameters as well as in the positional data by our model were reasonable. In all cases, the (known) movement parameters in the four realistic simulation settings were properly retrieved. Only when the observational time-step considered were 30, 60 and 90 min the parameters were not well estimated, but only for certain movement trajectories characterized by large distance travelled per unit of time (*k*). This finding suggests that mobile fishes may experience relevant changes in both position and detection probability within the same time-step which may induce a bias in their movement path when large observational time-steps are considered. Therefore, a trade-off between how fast an individual moves and the duration of the time-step should be considered, both in the modelling process and in designing the array of receivers (i.e., between-array distance) as well as the periodicity at which acoustic pulses should be optimally emitted. To that end, pilot studies aiming at generating preliminary information about the movement pattern of a given species is highly advisable for optimizing the design of an acoustic array and the transmitter settings. The approach proposed by Pedersen et al. [39] or the R-code provided in the S1 Appendix should help for conducting simulation experiments for fulfilling this task.

The application of the Bayesian SSM to case study of the pearly razorfish revealed that the species’ activity is constrained within a very small circular home range with a *radius* of 145.04 ± 49.5 m (between-fish mean and s.d.) and that the exploration rate (*k*) of the home range was 0.005 ± 0.002 min^-1^. That is, despite that the pearly razorfish is abundant within a large area of connected sandy and soft bottom habitats, a given individual fish only uses a very small fraction of such large area of suitable habitat, at least at the temporal scale considered here (up to effective 15 tracking days). This pattern has been observed in other sequential hermaphrodites living in coastal areas of the Mediterranean. For example, the radius of the circular home range estimated for the Mediterranean rainbow wrasse, *Coris julis*, was only 227.6 m [14]. These findings confirmed that even relative small marine protected areas may provide a significant protection to the adult stock of the pearly razorfish [16–18]. Moreover, our methodological approach provides a tool to quantify the large variability of behavior between individual fishes fish. This is especially relevant when considering the growing evidence for the ecological and evolutionary role of between-fish variability in behavioral traits [76]. In fact, Alós et al. [77] demonstrated how among-individual variability in the home range behavior can generate selection gradients through harvesting individuals characterized by larger home range radius and large exploration rate (*k*) that, when combined, implies that fishes with faster swimming speeds are selectively harvested. SSM, therefore, provides a novel tool for future studies aimed at investigating the ecological and evolutionary consequences of the home range behavior in exploited fishes, with especial emphasis on the relationship among home range behavior and harvesting-induced selection [78].

Although useful as discussed, the Bayesian SSM proposed here has limitations too. First, it is a method based on a MCMC-algorithm, thus it is computation-intensive and reaching convergence for all the parameter may require long computation times [75] compared with frequentist solutions [52]. In our case, the real data-sets obtained for the pearly razorfish was however analysed in a reasonable computing time (~3 hours). Nevertheless, computation time may be a severe constraint our Bayesian approach when long time series (months or years) are available. The recent approach suggested by Albersten et al. [12] implemented in the novel R-package Template Model Builder (TMB) for fitting SSM models to movement data provides a promising tool to alleviate MCMC computational costs in the future when applying the methods presented in this paper. Second, our approach is currently underexploiting some of the potential benefits of SSM when applied to movement data. SSM is a specific case of a family of Hidden Markov Models (HMM) where data (in our case positional data) are observed with uncertainty [40]. HMM are becoming popular for understanding fine-scale animal behavior in reality mining applications because of their ability to differentiate different behavioral states from movement data [23,79]. One interesting application has been proposed by Patterson et al. [80] who demonstrated the usefulness of the method for discriminating if a given fish is in a resident or migratory state using electronic tagging data in the southern bluefin tuna (*Thunnus maccoyii*). Therefore, as it was suggested in the theoretical framing of this manuscript, the Bayesian SSM can be readily expanded for discriminating different behavioral states such as foraging, hunting or spawning. Third, random effects can also be readily incorporated into hierarchical Bayesian models to facilitate the convergence of the model parameters where the individual movement parameters are estimated from the individual-level data but they are assumed to be distributed around a population mean [81]. All three limitations maybe solved in the future to improve the performance of the application of Bayesian SSM to acoustic tracking data.

To conclude, the use of SSMs opens new possibilities for analysing movement data of any animal, including of course sedentary marine fishes, which can provide novel insights for improving our understanding of home range behavior. Fish path and movement parameters can also be used for deriving biologically relevant information with direct application for promoting the sustainability of exploited fish and for providing better understanding of relevant spatial ecological processes. The approach demonstrated here is flexible to include different movement processes and can be easily adapted to acoustic tracking study with other receiver configuration arrays. Similarly, a wide range of behavioral models can be analysed, increasing the potentials of biotelemetry as recently suggested by Krause et al. [23], Donaldson et al. [28] and Hussey et al. [27].

## Supporting Information

S1 Appendix R-Code for fitting the Bayesian state-space model. Simulation code to generate acoustic tracking data and to estimate the movement parameters (home range behavior) and positions using a Bayesian state-space model.
(DOC)

S1 Dataset Temporal series of acoustic detections of the six individuals of pearly razorfish, *Xyrichtys novacula* tracked in 2011 and coordinates (in UTM) of the array of omnidirectional receivers located in the waters of Mallorca Island (NW Mediterranean). (ZIP)
