# Supplementary material for: Bayesian State-Space Modelling of Conventional Acoustic Tracking Provides Accurate Descriptors of Home Range Behavior in a Small-Bodied Coastal Fish Species
Source: PLoS One. 2016 Apr 27;11(4):e0154089. doi: 10.1371/journal.pone.0154089 (PMC4847866; doi:10.1371/journal.pone.0154089)
Supplement: S1 Appendix — Simulation code to generate acoustic tracking data and to estimate the movement parameters (home range behavior) and positions using a Bayesian state-space model. (DOC) [file pone.0154089.s001.doc]

Supporting Information for the manuscript (S1 Appendix):

**Bayesian state-space modelling of conventional acoustic tracking provides accurate descriptors of home range behavior in a sedentary coastal fish species**

By

Josep Alós, Miquel Palmer, Salvador Balle & Robert Arlinghaus

Contents:

**R-Code** Estimation of the movement parameters (home range behavior) and positions using a State-space model in acoustic tracking

#-------------------------------------------------------------------------

# Supplementary material of the paper:

# Alós, Palmer, Balle & Arlinghaus (last update: April 2016)

# Bayesian state-space modelling of conventional acoustic tracking provides accurate descriptors #of home range behavior in a sedentary coastal fish species

#

# R-code for simulating and analyzing the detection pattern produced by a fixed array of receivers

# Structure of the R-code

# 1) Simulating the trajectory of a fish moving according a HR behavior [1]

# 2) Simulating the detection pattern obtained by a conventional acoustic tracking experiment (inspired in [2])

# 3) Estimating the movement parameters and the fish trajectory using a Bayesian State-Space model approach

# 4) Inspecting the results and producing graphs

# 5) References ([#])

#-------------------------------------------------------------------------

# Removing any existing data and loading libraries

remove(list=ls())

library(R2jags)

library(ggplot2)

library(gridExtra)

#-------------------------------------------------------------------------

# 1) Simulating the trajectory of a fish that moves according to HR behavior

#-------------------------------------------------------------------------

# 1.1 Time step, detection binning and number of simulated days

dt = 60 # time-step in the simulation of the trajectory considered (seconds)

P = 30 # time-step for pooling acoustic detections (the number of beeps is summed over periods of P*dt seconds)

days = 12 # length of the time series (in days)

detmax = round(P*dt/60) # number of beeps emitted in P*dt seconds (1 beep/minute)

# 1.2 Movement parameters corresponding to a home range movement ([1])

# Center of the home range

HRx = 0 # Longitude (meters)

HRy = 0 # Latitude (meters)

HR = data.frame(HRx,HRy)

# k, radius, ep and SD (([1])

k<-0.01/60 # sec-1

radius = 387 # meters

ep = -radius^2*k/log(1-0.95) # Equation 7; meters^2 sec^-1

SD = sqrt(ep*(1-exp(-2*k*dt))/(2*k)) # Equation 6; meters

# 1.3 Simulating fish path (Latitude and Longitude in m) using equation 5 and 6

# 1.3.1 Moving the fish during one day for generating a randomly chosen tag-and-release position (inspired in [2])

xy = matrix(NA,1,2)

xy[1,1] = HRx[1] # fish starts moving in the center of the HR

xy[1,2] = HRy[1]

iterb = 1*14*60*60/dt # number of time steps in one day (14 hours of day-time)

temp = cbind(rnorm(iterb,0,SD),rnorm(iterb,0,SD)) # stochastic part of Equation 5

k1=exp(-dt*k) # speeding the loop

for (i in c(2:iterb)){

xy[1,1]=(k1*(xy[1,1]-HRx))+HRx+temp[i,1]

xy[1,2]=(k1*(xy[1,2]-HRy))+HRy+temp[i,2]

}

xy0=xy

xy0 # tag-and-release position

# 1.3.2 Simulating a fish trajectory for iter time steps starting at the tag-and-release position

iter = days*14*60*60/dt # number of time steps in one day (14 hours of day-time)

xy = matrix(NA,iter,2) # template for fish positions

xy[1,1] = xy0[1,1] # fish starts moving at the tag-and-release position

xy[1,2] = xy0[1,2]

for (i in c(2:iter)){

temp = rnorm(2,0,SD)

xy[i,1] = (k1*(xy[(i-1),1]-HRx))+HRx+temp[1] # Equation 5 (longitude)

xy[i,2] = (k1*(xy[(i-1),2]-HRy))+HRy+temp[2] # Equation 5 (latitude)

}

# Saving positions and temporal series as a frame for analysis and visualization

temp=seq(as.Date("2011/08/01"),11+as.Date("2011/08/01"),1)

day=rep(temp,each=14*60*60/dt)

XY<-data.frame(xy,day)

colnames(XY) = c("x","y","Day")

# Cheking the radius (% of positions outside the radius should be around 5%)

#100*sum((XY$x^2+XY$y^2)^(1/2)>radius)/iter

# Averaged position at each period of P time steps

iterP = round(iter/P) # length of the series

x = array(NA,iterP) # position (x coordinate; longitude)

y = array(NA,iterP) # position (y coordinate; latitude)

dayP = as.Date(array(NA,iterP)) # day

for (i in 1:iterP){

x[i] = mean(xy[(1+P*(i-1)):(P*i),1])

y[i] = mean(xy[(1+P*(i-1)):(P*i),2])

dayP[i] = as.Date(day[1+P*(i-1)])

}

# Save positions and temporal series as a frame for analysis and visualization

XYP<-data.frame(x,y,dayP)

colnames(XYP)<-c("x","y","Day")

head(XYP)

# Swimming speed (meters in P*dt seconds)

speed=sqrt((XYP[1:(iterP-1),1]-XYP[2:iterP,1])^2+(XYP[1:(iterP-1),2]-XYP[2:iterP,2])^2)

mean(speed)

range(speed)

#hist(speed)

# Swimming speed (meters in dt seconds)

speed=sqrt((XY[1:(iter-1),1]-XY[2:iter,1])^2+(XY[1:(iter-1),2]-XY[2:iter,2])^2)

mean(speed)

range(speed)

#hist(speed)

#-------------------------------------------------------------------------

# 2) Simulating an standard acoustic tracking experiment

#-------------------------------------------------------------------------

# 2.1 Deployment of an array omnidirectional acoustic receivers ([3])

# Emulating the experimental settings in [2]: 25 receivers were deployed at 300 m each

rx = rep(seq(HRx-600,HRx+600,300),each=5) # longitude

ry = rep(seq(HRy-600,HRy+600,300),5) # latitude

SUR = data.frame(rx,ry) # position of all receivers

colnames(SUR) = c("x","y")

R=length(rx) # number of receivers

# 2.2 Plot the acoustic array and the trajectory (all plots are print in point 5)

p1 = ggplot(legend=FALSE)+

coord_equal()+labs(title="Simulated fish")+

xlab("Longitude (m)") + ylab("Latitude (m)")+

geom_path(data=XY, aes(x,y,group=Day),color="blue")+ # positions at each dt

geom_path(data=XYP, aes(x,y,group=Day),color="red")+ # positions at each P

geom_point(data=SUR, aes(x,y),col="black")+ # receivers array

geom_point(data=HR, aes(HRx,HRy),col="green",size=I(3)) # HR center

p1

# 2.3 Probability of detection as a function of the distance between fish and receiver

# Distance-dependence was assumed to be sigmoid (Equation 8; [4] and [5]).

# However, the parameters of the sigmoid response (alpha and beta) may vary in time

# because they are environment dependent (e.g. tide effects [4])

# Here (as suggested in [2]) we assume that alpha and beta are constant at

# the within-day scale but change at the between-day scale

# Between-day variability of alpha and beta is known because a control tag were moored

# in the middle of the array.

#######This part of the code should be adapted to case study specificities##########

# Simulation a sequence of alpha and beta

inf = c(175,25) # (inflexion point) empirical values (mean and between-day sd) [2]

bet = c(-0.007,0.0005) # (beta) empirical values (mean and between-day sd) [2]

beta = rep(NA,iter) # template for a list of day-specific beta values

alpha = rep(NA,iter) # template for a list of day-specific alpha values

betaP = rep(NA,iterP) # template for a list of time step specific betas

alphaP = rep(NA,iterP) # template for a list of time step specific aplhas

day.alpha = NULL

day.beta = NULL

#list of alphas and betas at iter scale

days_unique=unique(dayP)

for (i in 1:length(days_unique)){

temp = which(day==days_unique[i])

temp.beta = rnorm(1,bet[1],bet[2]) # beta values are assumed to be normally distributed

beta[temp] = temp.beta

temp.inf = rnorm(1,inf[1],inf[2]) # inf values are assumed to be normally distributed

temp.alp = -temp.inf*temp.beta # alpha values

alpha[temp] = temp.alp

day.alpha=c(day.alpha,temp.alp)

day.beta=c(day.beta,temp.beta)

}

#list of alphas and betas at iterP scale

for (i in c(1:iterP)){

temp=which(dayP==days_unique[i])

betaP[temp]=day.beta[i]

alphaP[temp]=day.alpha[i]

}

# 2.4 Plotting probability of detection against distance between fish and receiver

d = c(0:1500) # distance (d)

pro_sim = NULL # template for probabilities (p)

for (i in 1:days){

p = exp(day.alpha[i]+day.beta[i]*d)/(1+exp(day.alpha[i]+day.beta[i]*d))

group = rep(i,length(p)) # day label

pro_sim<-rbind(pro_sim,cbind(d,p,group))

#plot(d,p)

}

colnames(pro_sim) = c("d","p","date")

pro_sim<-as.data.frame(pro_sim)

p2=ggplot(pro_sim, aes(d, p,color=as.factor(date),group=as.factor(date)))+ geom_path()+

scale_x_continuous(limits=c(0,1000))+#limit of your plot

labs(title="Simulated between-days variability")+

xlab("Distance (m)") + ylab("Probability of detection")+

theme(legend.position = "none")

p2

# 2.5 Simulating the detection pattern

# REC is a matrix of detection or not (0 or 1) by each receiver at each dt

REC = array(NA,dim=c(iter,R))

for (i in 1:iter){

temp = rbind(xy[i,],cbind(rx,ry)) # Merging fish position and receivers position

d = as.matrix(dist(temp))[1,2:(length(rx)+1)] # Distance from the fish to each receiver

p = exp(alpha[i]+beta[i]*d)/(1+exp(alpha[i]+beta[i]*d)) # Detection probability

temp = NULL

for (j in 1:R){temp=c(temp,ifelse(runif(1,0,1)<p[j],1,0))} # Comparing p with a with a random value between 0 and 1 # when random number < p, then the fish is detected by the receiver

REC[i,] = temp

}

# Averaged number of receivers detecting a beep

mean(apply(REC,1,sum))

# RECP is a matrix of the number of detections by each receiver over the period P*dt seconds

iterP = round(iter/P)

RECP = array(NA,dim=c(iterP,R))

for (i in 1:iterP){

RECP[i,] = colSums(REC[(1+P*(i-1)):(P*i),])

}

# 2.6 Creates SUM: total detections (i.e., all receivers) over each period P*dt seconds

SUM = apply(RECP,1,sum)

# Saving SUM as a frame for analysis and visualization and plot

#SUM = data.frame(SUM,DATEP,dayP)

#colnames(SUM) = c("SUM","Date","Day")

SUM = data.frame(SUM,dayP)

colnames(SUM) = c("SUM","Day")

time<-seq(from=1,to=dim(SUM)[1],by=1)#creates a sequence of time-steps from 1

#plot

p3=ggplot(legend=FALSE)+

geom_area(data=SUM,aes(x=time,y=SUM),fill="blue")+

labs(title="Detections x P")+xlab("Date") + ylab("Detections")

p3

#-------------------------------------------------------------------------

# 3) Estimation of the movement parameters and positions using the SSM

#-------------------------------------------------------------------------

# 3.1 Input

data.jags = list(

dt = P*dt, # Duration of the period over which the number of detections were summed (seconds)

N = dim(RECP)[1], # Number of P periods

rx = rx, # Receivers position (longitude, m)

ry = ry, # Receivers position (latitude, m)

x0 = x[1], # Initial fish position (at tag-and-release) (longitude, m)

y0 = y[1], # Initial fish position (at tag-and-release) (latitude, m)

REC = RECP, # Detection matrix:

# CELLS: Number of detections over a given time period (P)

# COLUMNS: Receivers

# ROWS: Evenly distributed, ordered temporal series

detmax = detmax, # Maximum number of possible detections (=number of beeps emitted) over the period P

alpha = alphaP, # alpha value (day specific)

beta = betaP, # beta value (day specific)

R = R # Number of receivers

)

# 3.2 Initial values

# Initial values for each position is based in the weighted mean algorithm described in [6]

xyinterp = array(NA,dim=c(iterP,2))

xyinterp[1,]=xy0

for (i in 2:iterP){

xyinterp[i,1]= sum(RECP[i,]*rx)/sum(RECP[i,])

xyinterp[i,1]= ifelse(is.na(xyinterp[i,1]),xyinterp[(i-1),1],xyinterp[i,1])

xyinterp[i,2]= sum(RECP[i,]*ry)/sum(RECP[i,])

xyinterp[i,2]= ifelse(is.na(xyinterp[i,2])==TRUE,xyinterp[(i-1),2],xyinterp[i,2])

}

xyinterp[1,] = NA

#Initial values for the other parameters

inits = function(){list(

HRx = mean(na.exclude(xyinterp[,1])), # Position of the center of the HR (longitude, m)

HRy = mean(na.exclude(xyinterp[,2])), # Position of the center of the HR (latitude, m)

x = xyinterp[,1], # Fish position (longitude)

y = xyinterp[,2], # Fish position (latitude)

k = runif(1,0,0.01), # k value

radius = runif(1,50,500) # radius

)}

# 3.3 Model for JAGS

sink("model.txt")

cat("model {

#Detection probability

for (t in 2:N){ # number of P periods (rows of REC)

for (j in 1:R){ # Receivers (columns of REC)

REC[t,j]~dbin(p[t,j],detmax) # Expected number of detections (conditional to p and detmax)

logit(p[t,j])<-alpha[t]+beta[t]*d[t,j]

#d[t,j]<-sqrt((x[t]-rx[j])^2+(y[t]-ry[j])^2) # Fish:receiver distance

d[t,j]<-sqrt((mean(x[(t-1):t])-rx[j])^2+(mean(y[(t-1):t])-ry[j])^2) # Fish:receiver distance

}

}

#moving the fish (movement model)

#initial position

x[1]<-x0

y[1]<-y0

#successive positions

for (t in 1:(N-1)){ # number of P periods (rows of REC)

tempy[t+1]<-(exp(-k*dt)*(y[t]-HRy))+HRy # Equation 5 (longitude)

y[t+1]~dnorm(tempy[t+1],tau) # tau is the inverse of sd^2

tempx[t+1]<-(exp(-k*dt)*(x[t]-HRx))+HRx # Equation 5 (latitude)

x[t+1]~dnorm(tempx[t+1],tau)

}

tau<-(2*k)/(ep*(1-exp(-2*k*dt))) # Equation 6; tau is the inverse of sd^2

ep<--radius^2*k/log(1-0.95) # Equation 7

#priors

HRy~dnorm(0,10E-6)

HRx~ dnorm(0,10E-6)

k~dunif(0,1)#dgamma(0.01,0.01)

radius~dnorm(0,10E-6)

}",fill = TRUE)

sink()

#3.4 Settings and running JAGS

# Parameters monitored

params <- c("k","radius","HRx","HRy")#,"x","y")

# MCMC settings

ni <- 5000 # Number of iterations

nt <- 10 # Thinning (one out nt iterations will be kept)

nb <- 10000 # Number of burning iterations

nc <- 3 # Number of chains

results = jags(data.jags, inits, params, "model.txt", n.chains = nc,

n.thin = nt, n.iter = ni, n.burnin = nb,jags.seed = 123)

pre.time = Sys.time()

results = update(results , n.iter=100, n.thin=5) # CAUTION!!!

post.time = Sys.time()

print(post.time-pre.time)

#Check Chains for convergence

traceplot(results, varname = c("k"))

abline(h=k,lwd=3)

traceplot(results, varname = "radius")

abline(h=radius,lwd=3)

traceplot(results, varname = "HRx")

abline(h=HRx,lwd=3)

traceplot(results, varname = "HRy")

abline(h=HRy,lwd=3)

#summary of results

results$BUGSoutput$summary

#-------------------------------------------------------------------------

# 4) References

#-------------------------------------------------------------------------

#1. Palmer M, Balle S, March D, Alós J, Linde M (2011) Size estimation of circular home range from fish mark-release-(single)-recapture data: case study of a small labrid targeted by recreational fishing. Marine Ecology-Progress Series 430: 87-97.

#2. Alós J, Cabanellas-Reboredo M, Lowerre-Barbieri S (2012) Diel behaviour and habitat utilisation by the pearly razorfish during the spawning season. Marine Ecology Progress Series 460: 207-220.

#3. Heupel MR, Semmens JM, Hobday AJ (2006) Automated acoustic tracking of aquatic animals: scales, design and deployment of listening station arrays. Marine and Freshwater Research 57: 1-13.

#4. How J, de LeStang S (2012) Acoustic tracking: issues affecting design, analysis and interpretation of data from movement studies. Marine and Freshwater Research 63: 321 - 324.

#5. Pedersen MW, Weng KC (2013) Estimating individual animal movement from observation networks. Methods in Ecology and Evolution 4: 920-929.

#6. Simpfendorfer C, Heupel M, Hueter R (2002) Estimation of short-term centers of activity from an array of omnidirectional hydrophones and its use in studying animal movements. Canadian Journal of Fisheries and Aquatic Sciences 59: 23 - 32.
